# Resident birds are more behaviourally plastic than migrants

**DOI:** 10.1038/s41598-022-09834-1

**Published:** 2022-04-06

**Authors:** Federico Morelli, Yanina Benedetti, Daniel T. Blumstein

**Affiliations:** 1 Faculty of Environmental Sciences, Czech University of Life Sciences Prague, Kamýcká 129, 165 00 Prague 6, Czech Republic; 2 Department of Ecology and Evolutionary Biology, University of California, Los Angeles, CA USA

**Keywords:** Ecology, Zoology, Ecology, Environmental sciences

## Abstract

Species subjected to more variable environments should have greater phenotypic plasticity than those that are more restricted to specific habitat types leading to the expectation that migratory birds should be relatively more plastic than resident birds. We tested this comparatively by studying variation in flight initiation distance (FID), a well-studied antipredator behaviour. We predicted that variation in FID would be greater for migratory species because they encountered a variety of locations during their lives and therefore had less predictable assessments of risk compared to more sedentary species. Contrary to our prediction, we found that non-migratory species (sedentary) had greater variation in FID than migratory ones. Migratory and partially migratory birds had greater average FIDs than sedentary birds, suggesting that they were generally more wary. These results suggest that the predictability associated with not migrating permits more nuanced risk assessment which was seen in the greater variation in FID of sedentary bird species.

## Introduction

Flight initiation distance (FID), a component of escape behaviour, is commonly used as a proxy of fear in animals and has provided fundamental insights into predator–prey interactions^1–5^. Fear is defined by ethologists as "a motivational state aroused by specific stimuli that give rise to defensive behaviour or escape"^6^. FID is considered a reliable metric quantifying the level of risk-taking in animals because it reflects the trade-off between costs of premature escape and benefits from staying^7,8^. Thus, FID indicates if individuals take more risk (i.e. delay their escape) or take less risk (i.e. escape earlier) when facing a potential threat^9^. Escape behaviour and longer times to take-off could be associated with increasing bird's vulnerability^10^.

FID is particularly well-studied in birds and many factors that influence escape have been identified (summarised in^11^). For instance, it is well known that the FID is strongly positively associated with a species’ body size since larger species need more time to get airborne and escape^12–15^. Additionally, changes in FID are associated with a variety of different factors (e.g. predator starting distance^16,17^, the density of potential predators^18^, the population density of the species^19^, flock size^3^, where individuals are along a rural–urban gradient^4^, season^20,21^ and other life-history traits as the type of diet^5^.

Most previous studies have focused on the average FID, but variation in FID could also be considered a measure of plasticity at the individual and population level^22–24^. These studies have shown that intraspecific variation in FID is associated with a greater variety of habitat use and is associated with a variety of life history traits suggesting that it is part of an antipredator/life history syndrome. At the intraspecific level, diel variation in FID may be associated with foraging success. We extend these previous insights by focusing on the coefficient of variation in FID as a measure of behavioural plasticity for avian species. We then asked how this measure of variation was associated with whether or not a species was migratory or not. The migratory behavior of birds is a regular behaviour involving billions of individuals, which travel across diverse regions of the planet^25,26^. Migratory birds are quantitatively different than non-migratory species in several ways. For instance, migratory birds face a thermal niche change between breeding and non-breeding seasons^26^, and they tend to have smaller brains than sedentary species^27^. We expected that migratory species would be significantly more plastic in their FID responses. This was based on the assumption that they must experience more habitat types and less predictable risks during their lives compared with more sendintary species. To isolate this effect, we controlled for other variables that are known to explain variation in FID. Because previous studies have identified significant phylogenetic signals in life history and escape behaviour, we compared, using AIC (Akaike's information criterion), models that did or did not incorporate phylogenetic information.

## Results

A total of 1240 FID observations were collected in USA, while 2359 observations were collected in Australia from species with complete information on the set of our independent variables, with 115 observations recorded for two bird species present in both countries (Table S1). Overall, 539 observations were collected for migratory birds, 528 for partially migratory birds, while 2647 observations were collected for sedentary birds (Table S1). A total of 15 different migratory species, 9 partially migratory and 9 sedentary species were studied in the USA, while 2 migratory, 9 partially migratory and 49 sedentary species were studied in Australia (Tables S1 and S2).

**Figure 1:**
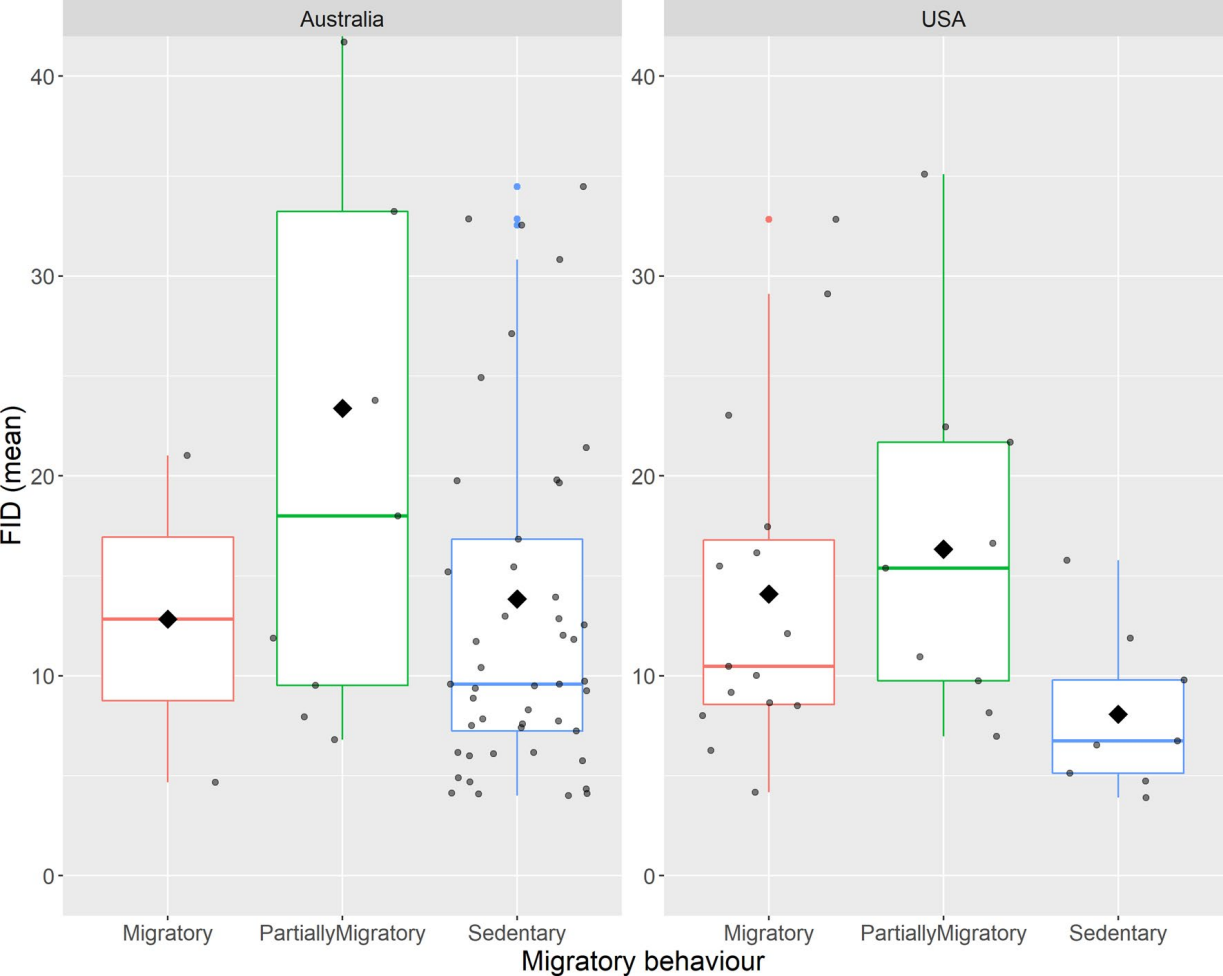
Difference in flight initiation distance (FID in metres) among migratory, partially migratory and sedentary birds, split by country where data were collected. The box plots show medians (horizontal bar), quartiles, 5- and 95-percentiles, jittered points (small-grey dots) and extreme values (small-coloured dots). Mean values are indicated with black rhombus.

**Figure 2:**
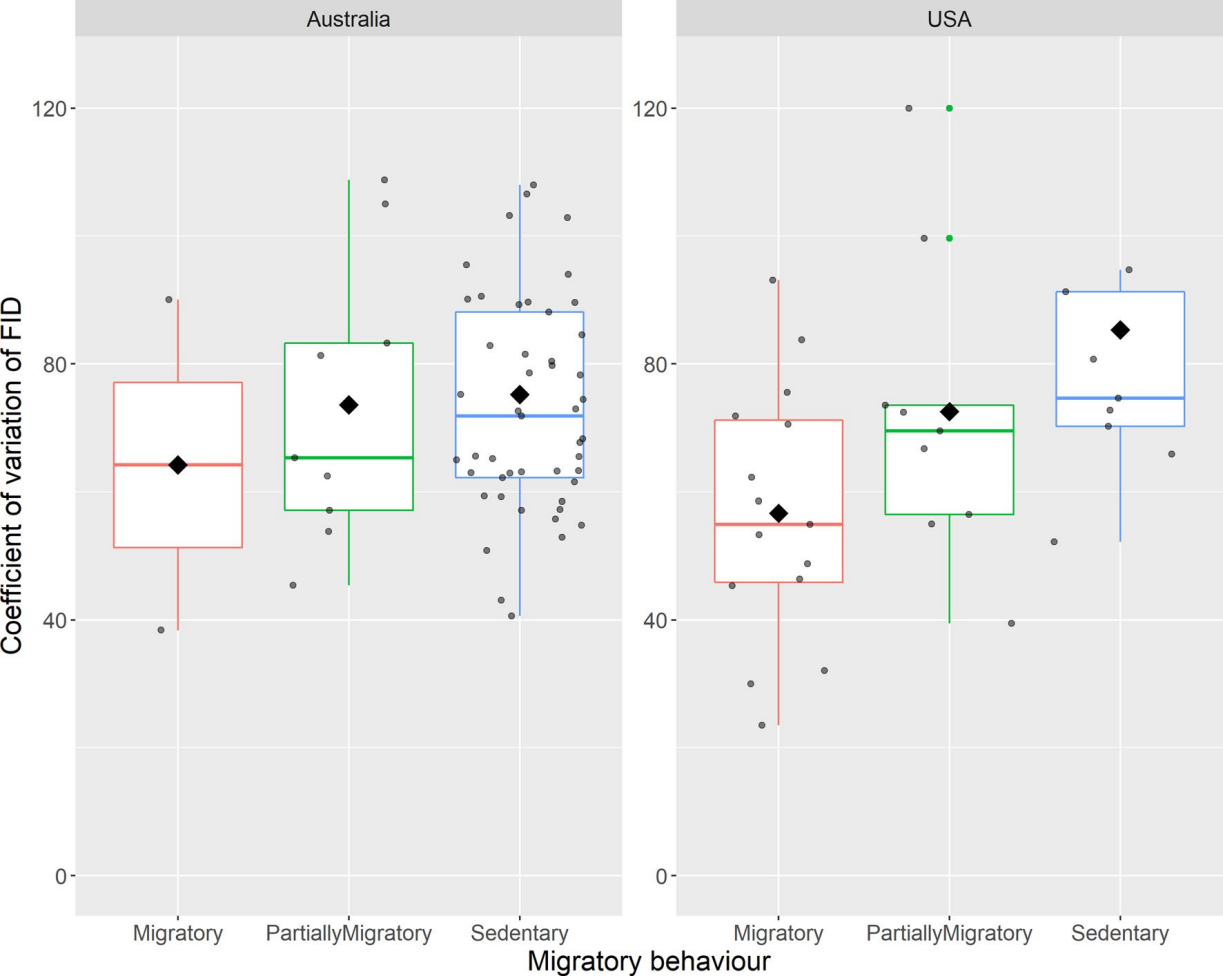
Difference in plasticity of escape behaviour (coefficient of variation of FID) migratory, partially migratory and sedentary birds, split by country where data were collected. The box plots show medians (horizontal bar), quartiles, 5- and 95-percentiles, jittered points (small-grey dots) and extreme values (small-coloured dots). Mean values are indicated with black rhombus.

The mean values of FID were higher for migratory and partially migratory birds than for sedentary species in USA, while in Australia partially migratory birds fled at the greatest distance (Table S1, Fig. 1). Plasticity was unrelated to the number of observations (Fig. S1), and was relatively higher in sedentary and partially migratory birds than in migratory species in USA and Australia (Table S1, Fig. 2). Of the top ten most plastic birds (Table S1, Fig. S2), 60% were classified as sedentary species, with 2 partially migratory species for USA (*Corvus corax* and *Molothrus ater*) and 2 for Australia (*Falco cenchroides* and *Elanus axillaris*)(Table S2). No migratory species were selected in the top ten most behaviorally plastic birds (Table S2).

We found no significant phylogenetic signal in behavioural plasticity using any of the phylogenetic signal statistics, while the phylogenetic signal was significant for the other life-history traits analysed (Table 1). Any way analysed, we found a consistent result: there was a significantly positive association between plasticity and being sedentary, and partially migratory when compared with migratory birds (Table 2). The non-phylogenetic model had a substantially lower AIC (delta AIC = 50.5). Interestingly, the phylogenetic model also suggested that longer-lived bird species have greater behavioural plasticity (Table 2) while the other explored variables were not statistically significant (Table 2, Figs. S3 and S4).

**Table 1:** Results of phylogenetic signal of escape behavior plasticity (coefficient of variation of FID) and other life history traits of the 95 bird species included in this study.

Variables	Abouheif’s		I		K		K *		Lambda	
	Stat	*p*	Stat	*p*	Stat	*p*	Stat	*p*	Stat	*p*
Relative variance of FID	0.037	0.216	− 0.0005	0.129	0.288	0.223	0.336	0.279	0.174	0.589
Habitat breadth	**0.244**	**0.002**	**0.019**	**0.006**	**0.441**	**0.011**	**0.533**	**0.003**	**0.821**	**0.001**
Diet breadth	**0.333**	**0.001**	**0.031**	**0.002**	**0.393**	**0.002**	**0.440**	**0.006**	**0.699**	**0.001**
Body mass	**0.288**	**0.010**	**0.0260**	**0.009**	**0.942**	**0.002**	**0.727**	**0.008**	**0.857**	**0.001**
Life span max	**0.399**	**0.001**	**0.0369**	**0.002**	**0.465**	**0.022**	**0.565**	**0.012**	**0.791**	**0.001**

The table shows Abouheif’s Cmean, Moran’s I, Blomberg’s K and K*, and Pagel’s Lambda statistics (Stat) and associated p values for each value. Significant variables are highlighted in bold.

**Table 2:** Results of models explaining plasticity in escape behaviour in birds as a function of starting distance, the number of observations (FID count), habitat and diet breadth, migratory behaviour (migratory, partially migratory and sedentary birds), body mass, life span and diet type of species, considering also the country where data were collected.

Variable/Model	Estimate	2.50%	97.50%	SE	t value	*p*
**Model 1 (GLM)**						
Intercept	59.237	35.910	82.564	11.902	4.977	< 0.001
Starting distance (mean)	− 0.208	− 0.545	0.130	0.172	− 1.206	0.231
FID (count)	− 0.032	− 0.161	0.098	0.066	− 0.479	0.633
Habitat breadth	0.054	− 3.146	3.254	1.633	0.033	0.974
Diet breadth	− 49.251	− 148.363	49.862	50.569	− 0.974	0.333
Migration: partially migratory	**17.323**	**0.366**	**34.280**	**8.652**	**2.002**	**0.048**
Migration: sedentary	**21.238**	**4.858**	**37.618**	**8.357**	**2.541**	**0.013**
Body mass	− 0.001	− 0.008	0.006	0.004	− 0.218	0.828
Life span (max)	0.225	− 0.113	0.563	0.172	1.306	0.196
Diet: Omnivore	− 1.280	− 15.719	13.160	7.367	− 0.174	0.863
Diet: Plant/seed/nectar	7.277	− 6.387	20.942	6.972	1.044	0.300
Diet: Vertebrates	13.766	− 6.926	34.458	10.557	1.304	0.196
Country: Australia/USA	0.500	− 35.091	36.091	18.159	0.028	0.978
Country: USA	4.084	− 9.037	17.205	6.695	0.610	0.544
**Model 2 (PGLS)**						
Intercept	51.702	1.917	101.486	25.401	2.035	0.045
Starting distance (mean)	0.120	0.014	0.227	0.054	2.216	0.030
FID (count)	− 0.195	− 0.692	0.302	0.254	− 0.768	0.445
Habitat breadth	0.681	− 3.127	4.488	1.943	0.350	0.727
Diet breadth	− 27.274	− 134.606	80.058	54.762	− 0.498	0.620
Migration: partially migratory	**21.136**	**6.421**	**35.851**	**7.508**	**2.815**	**0.006**
Migration: sedentary	**19.233**	**2.784**	**35.681**	**8.392**	**2.292**	**0.025**
Body mass	− 0.005	− 0.015	0.004	0.005	− 1.083	0.282
Life span (max)	**0.569**	**0.106**	**1.032**	**0.236**	**2.408**	**0.018**
Diet: Omnivore	− 4.052	− 20.566	12.462	8.426	− 0.481	0.632
Diet: Plant/seed/nectar	12.432	− 3.351	28.215	8.053	1.544	0.127
Diet: Vertebrates	12.796	− 13.941	39.532	13.641	0.938	0.351
Country: Australia/USA	− 20.829	− 54.873	13.216	17.370	− 1.199	0.234
Country: USA	− 5.248	− 21.005	10.509	8.040	− 0.653	0.516

The model 1 is a generalized linear model while the model 2 is a phylogenetic generalized least squares model (PGLS) incorporating a phylogenetic correlation term among bird species. Models are based on 3714 observations of FID collected in USA (1240 records), Australia (2359 records) and both (115 records) for 95 bird species. The table shows the values of estimates, the lower (2.5%) and upper (97.5%) limits of confidence intervals, standard error (SE), t and p values. Significant variables are highlighted in bold. Model 1 AIC: 863.93. Model 2 AIC: 914.40.

## Discussion

Using the coefficient of variation of FID, a potentially species-specific value that is independent of the FID range and/or a species’ body size, we found that sedentary and partially migratory birds were comparatively more plastic in their escape behaviour than migratory species. This was contrary to our expectation, but we speculate that sedentary and partially migratory birds, that spend their entire life cycle in the same habitat or in a relatively narrow distribution range than migratory species, benefit from more variable and perhaps nuanced responses to threats. Interestingly, migratory and partially migratory birds had relatively longer escape responses relatively than sedentary species in USA, while such differences were less clear in Australia, where partially migratory species had longer average FID’s. Greater wariness could be associated with the relatively smaller brain that characterised the migratory birds if compared to the sedentary species^27^. A smaller brain in migratory birds could be an energy saving strategy for species’ that have relatively high energetic needs associated with a highly mobile life cycle^27^. If generally true in other species, this suggests that migratory birds may be particularly vulnerable to human disturbance.

While there are significant effects of a species’ evolutionary history on specific traits, our non-phylogenetic model was a more efficient model than the phylogenetic one suggesting that the statistical relationship between migration and plasticity is not influenced by evolutionary relationships between species. Interestingly, in the phylogenetically-informed analysis, we also found a positive and significant association between behavioural plasticity and species' life span (Fig. S5) that was independent of migratory status: longer-lived species were more plastic in their escape responses. While this makes sense, we are unable to explain why the result did not emerge from the better-supported non-phylogenetic analysis.

A shortcoming of our study is that we assigned migratory status at the species level, but there is intraspecific variation in migratory behavior in some species^28^. If anything, this would make it more difficult to detect the effect of migration on variable escape responses. Any classification of birds as “migratory”, “partially migratory” and “non-migratory” species could be considered too reductionist because there are sedentary species that live within the same home ranges throughout the calendar year, while other birds shift subtly their environments among seasons within the same geographic area. Furthermore, there are short-distance migrants that may experience relatively similar threat environments because the assemblage of predators may not differ substantially from summer to winter, and finally long-distance migrants that potentially could experience very different threat among seasons. However, the notion that migrants need to be adapted to a wider range of dangers across their annual cycle remains to be demonstrated. In fact, some studies indicate that long-distance migratory species tend to select mostly similar environments throughout their annual cycle^29^, which may reduce the variation in predation risk between sedentary and migratory birds.

Finally, our findings suggest that migratory species may be more vulnerable to human disturbance than non-migratory species because of their increased wariness and less flexible response to threats. This suggests that future work studying variation in tolerance to disturbance in migratory and non-migratory species would be valuable^30^. If migratory behaviour were also associated with reduced tolerance to humans, this would further strengthen the need to protect migratory species from human disturbance.

## Methods

### Escape behaviour data collection

Flight initiation distance (FID) is defined as the distance at which animals take flight from approaching threats^31,32^. Following standardised methods^5^, trained observers identified a non-alarmed bird (i.e. resting or foraging) and directly approached at a measured pace of 0.5 m/s. The observer dropped a flag where they started the approach, a second flag where the bird moved its head to look in response to the approach, and a third flag where the bird began to escape. Escapes included both walking or running away and flying away. A final flag was dropped at the initial position of the bird when the experimental approach began. From these, the observer measured (with a meter tape, rangefinder, or a well-calibrated pace) the starting distance (first flag to bird’s initial location), alert distance (second flag to bird’s initial location), flight-initiation distance (third flag to bird’s initial location). The data were collected in a variety of habitats along the East coast of Australia as well as some other locations in Southern and Western Australia, central Colorado near Crested Butte, Colorado, and in Los Angeles, and Orange Counties in Southern California. Most data were collected in protected areas and reserves where the species were not harassed by humans, during the period 2000–2003.

### Bird life-history traits and measuring a proxy of behavioural plasticity

For each bird species, we collected the following life history data. Species body mass and habitat breadth were extracted from^33^ while diet breadth, life span (max) were extracted from^34^ and migratory status (resident or sedentary, partially migratory or migratory) was extracted from^35^. The main type of diet for each species was extracted from^36^.

We calculated the mean, maximum, minimum and standard devition of flight initiation distance for each bird species with > 5 observations, to avoid potential overinflated standard deviation estimates from species with only a few observations^9^. Then, for each species, we calculated our metric of plasticity based on the coefficient of variation (CV) of FID,

$$CV_{FID} = \frac{{FID_{SD} *100}}{FID}.$$

Because this is a ratio of the variance to the mean, plasticity is independent of body size.

### Statistical analyses

Life-history traits, including FID, often have a strong phylogenetic signal^37,38^. To create a phylogeny of the 95 species we had data on, we downloaded 1000 phylogenetic trees with the avian phylogenetic relationships from ‘www.birdtree.org’, selecting the backbone tree based on Ericson et al.^39^. The consensus tree was obtained applying the 50% majority rule (i.e. the proportion of a split to be present in all trees). We used the following R packages: ‘ape’^40^, ‘phangorn’^41^ and ‘Rphylip’^42^ to manipulate trees. With these trees, we tested the strength and significance of the phylogenetic signal^43^ for our metric of plasticity using all available indices in the package ‘phylosignal’ for R^44^: Abouheif’s Cmean^45,46^, Moran’s I^46^, Blomberg’s K statistic and statistic K* ^47^, and Pagel’s Lambda^48^.

To formally test the relationship between migratory behaviour and plasticity, we compared models with and without phylogenetic information and used AIC to identify the more efficient models to interpret^49^. First, we fitted a Generalized Linear Model^50^. Plasticity (CV_FID_) was modelled as a function of the following fixed effects: mean starting distance (included because it often explains substantial variation in FID^16^), the number of FID observations (to account for variation in our certainty of estimates), habitat breadth, diet breadth, whether a species are mainly sedentary, partially migratory or migratory, body mass, and maximum longevity. We included also country (USA or Australia) and diet type (classified as plant/seed/nectar, invertebrates, vertebrates or omnivore) as fixed effects to account for potential effects on variation in FID related to the region where data were collected and to different strategies of foraging. Then, we fitted a Phylogenetic Generalized Least Squares (PGLS) model with the same list of fixed effects but including also the phylogeny, and assumed a Brownian motion model of evolution (i.e. K < 1)^47^. The models were fitted by maximum likelihood, using the package “nlme” for R^51^. A test to detect potential multicollinearity issues among predictor variables was performed using the test of variance inflation factors (VIF) on the full generalized linear model, using the package ‘fmsb’ for R^52^. All variables had VIF < 3 and then were modelled together. The overall value of VIF for the full model was 1.21, suggesting no strong multicollinearity.

All statistical tests were performed using the R software^53^. The confidence interval for each variable was estimated by the Wald method from the package ‘MASS’^54^.

### Ethics

By design this work was minimally invasive. Research protocols were approved by the Macquarie University Animal Research Committee (99,021), the Rocky Mountain Biological Laboratory, and the UCLA Animal Research Committee (2000–147-01). Permission to work on land was acquired when required.

## Supplementary Information

Supplementary Information.

## Data Availability

The dataset used in this study is provided in the Electronic Supplementary Material, Table S2 and also published in Dryad Digital Repository. Dryad https://doi.org/10.5061/dryad.ns1rn8pv3. Additional information regarding the raw data can be provided directly by the authors, under reasonable request.
