# Supplementary material for: Resident birds are more behaviourally plastic than migrants
Source: Sci Rep. 2022 Apr 6;12:5743. doi: 10.1038/s41598-022-09834-1 (PMC8986783; doi:10.1038/s41598-022-09834-1)

**Supplementary material**

**“Resident birds are more behaviourally plastic than migrants”**

Table S1. Descriptive statistics for starting distance (mean), FID (mean, min and max values), behavior plasticity (coefficient of variation of FID), total number of observations and number of species, considering the country where data was collected and the type of migratory behaviour of birds. Additional details regarding the variables focused in the study for each bird species are provided in the Table S2. Total of observations 3714.

| **Type of behaviour** | **Country** | **Starting Distance (mean)** | **FID (mean)** | **mean FID (min)** | **mean FID (max)** | **Behavior plasticity (mean)** | **No. Observations** | **No. Species** |
| --- | --- | --- | --- | --- | --- | --- | --- | --- |
| Migratory |  | 27.528 | 15.161 | 4.180 | 35.818 | 57.248 | 539 | 18 |
|  | Australia | 20.732 | 12.844 | 4.669 | 21.018 | 64.207 | 40 | 2 |
|  | Australia/USA | 63.454 | 35.818 | 35.818 | 35.818 | 51.891 | 67 | 1 |
|  | USA | 26.039 | 14.093 | 4.180 | 32.830 | 56.678 | 432 | 15 |
| PartiallyMigratory |  | 38.452 | 19.493 | 6.808 | 57.626 | 72.479 | 528 | 19 |
|  | Australia | 43.630 | 23.388 | 6.808 | 57.626 | 73.606 | 198 | 9 |
|  | Australia/USA | 27.261 | 12.828 | 12.828 | 12.828 | 61.894 | 48 | 1 |
|  | USA | 34.517 | 16.339 | 6.967 | 35.086 | 72.527 | 282 | 9 |
| Sedentary |  | 27.940 | 12.949 | 3.909 | 46.795 | 76.773 | 2647 | 58 |
|  | Australia | 30.148 | 13.843 | 4.007 | 46.795 | 75.206 | 2121 | 49 |
|  | USA | 15.920 | 8.082 | 3.909 | 15.774 | 85.308 | 526 | 9 |

Table S2. Estimated values for starting distance, FID, behavior plasticity (coefficient of variation of FID), total number of observations (FID count), considering all data collected. Values of starting distance and FID are expressed as mean, min, max and standard deviation values. The table shows also the country where the data on FID were collected, and some traits for each bird species as body mass, main type of diet classified as a categorial variable, life span (max values recorded for the species), type of migratory behaviour of species, habitat and diet breadth. Life-history traits were extracted from the following published dataset: Sayol et al. 2018, 2020; Tobias & Pigot 2019 and Wilman et al. 2014. Species are ordered by migratory behavior and then by decreasing values of relative variance of FID. More details are provided in the methods section of the manuscript.

| **Species** | **Starting Distance (mean)** | **Starting Distance (sd)** | **Starting Distance (min)** | **Starting Distance (max)** | **FID (mean)** | **FID (sd)** | **FID (min)** | **FID (max)** | **FID (count)** | **Behavior plasticity (mean)** | **Dif SDFID** | **Country** | **Body mass (g)** | **Main diet** | **Lifespan max** | **Migratory Behaviour** | **Habitat Breadth** | **Diet Breadth** |
| --- | --- | --- | --- | --- | --- | --- | --- | --- | --- | --- | --- | --- | --- | --- | --- | --- | --- | --- |
| *Falcunculus frontatus* | 9.660 | 6.085 | 4.4 | 20 | 4.100 | 7.323 | 0 | 16.9 | 5 | 178.616 | 5.560 | Australia | 28.020 | invertebrates | 12 | Sedentary | 3.694 | 0.071 |
| *Picoides pubescens* | 16.450 | 13.503 | 4.2 | 40.8 | 5.117 | 8.462 | 0 | 21.5 | 6 | 165.391 | 11.333 | USA | NA | invertebrates | 12 | Sedentary | 3.367 | 0.073 |
| *Alectura lathami* | 26.611 | 20.320 | 8 | 94.9 | 12.037 | 13.000 | 0 | 63.5 | 27 | 107.999 | 14.574 | Australia | 2331.530 | plant/seed/nectar | 21 | Sedentary | 3.449 | 0.150 |
| *Trichoglossus haematodus* | 26.064 | 12.397 | 14.6 | 55.5 | 8.300 | 8.844 | 0 | 25 | 11 | 106.549 | 17.764 | Australia | 116.400 | plant/seed/nectar | 38 | Sedentary | 4.490 | 0.056 |
| *Cacatua galerita* | 27.856 | 17.607 | 5.4 | 91 | 15.450 | 15.947 | 0 | 76 | 36 | 103.216 | 12.406 | Australia | 721.965 | plant/seed/nectar | 120 | Sedentary | 2.619 | 0.136 |
| *Dacelo novaeguineae* | 27.866 | 17.649 | 3.8 | 88 | 12.540 | 12.898 | 0 | 59 | 53 | 102.855 | 15.326 | Australia | 337.063 | invertebrates | 29 | Sedentary | 3.841 | 0.026 |
| *Chenonetta jubata* | 42.843 | 29.627 | 6.9 | 160 | 21.420 | 20.456 | 1.8 | 108.5 | 78 | 95.499 | 21.424 | Australia | 809.980 | plant/seed/nectar | 18 | Sedentary | 4.510 | 0.000 |
| *Melanerpes formicivorus* | 26.472 | 11.694 | 9.2 | 58.22 | 11.885 | 11.254 | 0 | 47.57 | 74 | 94.695 | 14.587 | USA | 79.335 | plant/seed/nectar | 17 | Sedentary | 4.499 | 0.187 |
| *Geopelia humeralis* | 43.040 | 19.140 | 15.3 | 69.3 | 12.980 | 12.199 | 0 | 32.8 | 5 | 93.980 | 30.060 | Australia | 128.535 | plant/seed/nectar | 12 | Sedentary | 3.739 | 0.037 |
| *Carduelis tristis* | 17.226 | 11.786 | 5.4 | 43.8 | 9.786 | 8.931 | 2.3 | 41.5 | 21 | 91.263 | 7.440 | USA | 12.790 | plant/seed/nectar | 13 | Sedentary | 4.257 | 0.180 |
| *Fulica atra* | 25.063 | 20.012 | 6.2 | 66.2 | 19.763 | 17.896 | 3.3 | 53.8 | 8 | 90.557 | 5.300 | Australia | 746.060 | plant/seed/nectar | 32 | Sedentary | 8.026 | 0.160 |
| *Colluricincla harmonica* | 20.182 | 12.285 | 7.7 | 50 | 9.573 | 8.628 | 3.1 | 32.9 | 11 | 90.126 | 10.609 | Australia | 66.713 | invertebrates | 13 | Sedentary | 4.151 | 0.050 |
| *Menura novaehollandiae* | 18.950 | 15.845 | 5.4 | 69.3 | 10.409 | 9.332 | 3.6 | 50 | 22 | 89.654 | 8.541 | Australia | 976.285 | invertebrates | 26 | Sedentary | 2.376 | 0.057 |
| *Platycercus elegans* | 20.368 | 11.133 | 4.6 | 57.7 | 7.844 | 7.030 | 0 | 36.9 | 68 | 89.619 | 12.523 | Australia | 126.805 | plant/seed/nectar | 27 | Sedentary | 3.364 | 0.154 |
| *Passer domesticus* | 20.609 | 14.066 | 6.2 | 49.2 | 11.718 | 10.464 | 2.7 | 37.7 | 11 | 89.296 | 8.891 | Australia | 27.105 | plant/seed/nectar | 23 | Sedentary | 6.718 | 0.071 |
| *Meliphaga lewinii* | 16.137 | 13.368 | 3.8 | 70 | 7.407 | 6.524 | 0 | 35 | 41 | 88.075 | 8.729 | Australia | 33.990 | plant/seed/nectar | 12 | Sedentary | 4.952 | 0.090 |
| *Rhipidura leucophrys* | 20.561 | 14.305 | 4.6 | 82 | 11.825 | 9.994 | 2.3 | 62 | 51 | 84.509 | 8.735 | Australia | 27.220 | invertebrates | 9 | Sedentary | 4.445 | 0.000 |
| *Corvus coronoides* | 41.936 | 26.658 | 8.6 | 165 | 24.924 | 20.649 | 0 | 143 | 66 | 82.848 | 17.012 | Australia | 642.150 | omnivore | 22 | Sedentary | 5.416 | 0.138 |
| *Cracticus torquatus* | 42.050 | 25.145 | 8.5 | 70 | 19.800 | 16.137 | 4.6 | 48 | 6 | 81.498 | 22.250 | Australia | 81.778 | omnivore | 16 | Sedentary | 5.089 | 0.076 |
| *Callipepla californica* | 21.046 | 13.683 | 3.8 | 67.7 | 15.774 | 12.735 | 1.5 | 66.9 | 48 | 80.730 | 5.272 | USA | 168.600 | plant/seed/nectar | 10 | Sedentary | 4.506 | 0.125 |
| *Malurus lamberti* | 9.176 | 6.196 | 3.1 | 29.2 | 4.124 | 3.315 | 0.8 | 16.2 | 37 | 80.370 | 5.051 | Australia | 8.095 | invertebrates | 10 | Sedentary | 1.872 | 0.155 |
| *Acanthiza pusilla* | 9.430 | 4.542 | 3.1 | 20 | 4.007 | 3.196 | 0.8 | 17.7 | 27 | 79.741 | 5.422 | Australia | 7.610 | invertebrates | 17 | Sedentary | 4.732 | 0.099 |
| *Pelecanus conspicillatus* | 77.969 | 54.770 | 15.4 | 300 | 32.545 | 25.584 | 1 | 132.1 | 65 | 78.613 | 45.425 | Australia | 5478.750 | vertebrates | 25 | Sedentary | 5.360 | 0.019 |
| *Rhipidura fuliginosa* | 13.217 | 7.027 | 4.6 | 33.6 | 6.164 | 4.826 | 0 | 27 | 36 | 78.287 | 7.053 | Australia | 7.640 | invertebrates | 10 | Sedentary | 4.384 | 0.073 |
| *Ocyphaps lophotes* | 27.396 | 12.255 | 8 | 56.2 | 12.852 | 9.668 | 1.5 | 38.5 | 27 | 75.227 | 14.544 | Australia | 204.250 | plant/seed/nectar | 21 | Sedentary | 4.501 | 0.156 |
| *Chamaea fasciata* | 9.013 | 4.685 | 2.3 | 27.7 | 4.730 | 3.530 | 0 | 24.6 | 200 | 74.639 | 4.284 | USA | 14.715 | omnivore | 13 | Sedentary | 1.820 | 0.083 |
| *Neochmia temporalis* | 13.603 | 8.600 | 4.6 | 46 | 7.230 | 5.380 | 0 | 27.7 | 66 | 74.408 | 6.373 | Australia | 11.425 | plant/seed/nectar | 12 | Sedentary | 2.285 | 0.155 |
| *Gallinula tenebrosa* | 24.679 | 11.740 | 9.2 | 59.2 | 15.197 | 11.087 | 2 | 43.8 | 33 | 72.956 | 9.482 | Australia | 530.800 | plant/seed/nectar | 17 | Sedentary | 6.887 | 0.152 |
| *Carduelis pinus* | 13.017 | 4.682 | 7.6 | 21 | 8.250 | 6.002 | 2.5 | 18.5 | 6 | 72.755 | 4.767 | USA | 12.700 | plant/seed/nectar | 11 | Sedentary | 3.402 | 0.112 |
| *Phalacrocorax melanoleucos* | 56.539 | 32.602 | 15.4 | 161.5 | 19.664 | 14.281 | 4.2 | 65.1 | 67 | 72.625 | 36.875 | Australia | NA | omnivore | 12 | Sedentary | 7.380 | 0.020 |
| *Larus novaehollandiae* | 63.382 | 35.906 | 2.8 | 215.6 | 16.843 | 12.102 | 1 | 87.5 | 286 | 71.853 | 46.539 | Australia | 268.990 | omnivore | 15 | Sedentary | 5.717 | 0.132 |
| *Sitta carolinensis* | 14.256 | 9.495 | 3.1 | 34.6 | 3.909 | 2.745 | 0 | 7.81 | 11 | 70.210 | 10.347 | USA | 21.000 | omnivore | 10 | Sedentary | 2.894 | 0.162 |
| *Cracticus nigrogularis* | 23.220 | 14.015 | 13.1 | 46.2 | 7.740 | 5.286 | 1.8 | 13.8 | 5 | 68.290 | 15.480 | Australia | 127.325 | omnivore | 16 | Sedentary | 3.773 | 0.056 |
| *Zosterops lateralis* | 10.125 | 5.622 | 3.8 | 30.8 | 4.906 | 3.322 | 1.3 | 16.4 | 32 | 67.717 | 5.219 | Australia | 12.538 | omnivore | 13 | Sedentary | 4.541 | 0.100 |
| *Psaltriparus minimus* | 12.892 | 8.668 | 2.3 | 69.2 | 6.537 | 4.307 | 0 | 22.3 | 117 | 65.878 | 6.354 | USA | 5.300 | invertebrates | 9 | Sedentary | 5.071 | 0.073 |
| *Acanthorhynchus tenuirostris* | 9.628 | 4.379 | 3.8 | 19.2 | 4.685 | 3.072 | 0 | 13.1 | 39 | 65.585 | 4.944 | Australia | 10.114 | omnivore | 14 | Sedentary | 4.575 | 0.123 |
| *Egretta novaehollandiae* | 78.945 | 46.505 | 11.5 | 191.1 | 30.818 | 20.188 | 7 | 131.6 | 56 | 65.507 | 48.127 | Australia | 549.620 | omnivore | 20 | Sedentary | 13.920 | 0.027 |
| *Vanellus miles* | 92.182 | 48.442 | 21.4 | 211.4 | 46.795 | 30.510 | 6 | 163.1 | 60 | 65.198 | 45.387 | Australia | 383.000 | invertebrates | 17 | Sedentary | 3.951 | 0.000 |
| *Lichenostomus penicillatus* | 16.391 | 7.786 | 5.4 | 38.5 | 9.243 | 6.005 | 3.1 | 26.9 | 23 | 64.969 | 7.148 | Australia | 19.360 | invertebrates | 11 | Sedentary | 3.670 | 0.150 |
| *Porphyrio porphyrio* | 52.809 | 31.028 | 9 | 185.7 | 34.482 | 21.825 | 3 | 97.1 | 68 | 63.294 | 18.326 | Australia | 811.940 | plant/seed/nectar | 9 | Sedentary | 4.356 | 0.166 |
| *Ptilonorhynchus violaceus* | 16.404 | 6.899 | 4.6 | 27.1 | 8.880 | 5.618 | 3 | 21 | 25 | 63.261 | 7.524 | Australia | 215.955 | omnivore | 21 | Sedentary | 2.990 | 0.159 |
| *Platycercus eximius* | 20.000 | 10.918 | 6.2 | 49.2 | 9.504 | 5.997 | 0 | 22.9 | 24 | 63.095 | 10.496 | Australia | 104.000 | plant/seed/nectar | 37 | Sedentary | 4.036 | 0.154 |
| *Cacatua roseicapilla* | 48.746 | 36.442 | 8.5 | 191.43 | 7.591 | 4.778 | 0 | 23 | 122 | 62.945 | 41.155 | Australia | NA | plant/seed/nectar | 73 | Sedentary | 4.098 | 0.057 |
| *Sericornis frontalis* | 9.012 | 4.636 | 3.1 | 20.8 | 4.095 | 2.576 | 1.5 | 12.3 | 42 | 62.897 | 4.917 | Australia | 13.215 | invertebrates | 18 | Sedentary | 4.200 | 0.165 |
| *Threskiornis molucca* | 83.592 | 50.138 | 13.3 | 224 | 32.849 | 20.436 | 1 | 106.4 | 75 | 62.210 | 50.743 | Australia | NA | omnivore | 33 | Sedentary | 6.332 | 0.026 |
| *Alisterus scapularis* | 22.811 | 14.459 | 9.2 | 48.9 | 7.511 | 4.623 | 0 | 15.4 | 9 | 61.551 | 15.300 | Australia | 232.380 | plant/seed/nectar | 31 | Sedentary | 4.300 | 0.154 |
| *Threskiornis spinicollis* | 61.738 | 32.546 | 17.7 | 115.4 | 42.446 | 25.198 | 9.2 | 82.3 | 13 | 59.364 | 19.292 | Australia | 1349.048 | invertebrates | 39 | Sedentary | 3.620 | 0.024 |
| *Eopsaltria australis* | 16.156 | 8.285 | 3.1 | 44.6 | 9.369 | 5.549 | 2.3 | 25.5 | 84 | 59.222 | 6.787 | Australia | 20.200 | invertebrates | 13 | Sedentary | 4.789 | 0.000 |
| *Psophodes olivaceus* | 11.208 | 5.125 | 5.4 | 28.6 | 5.736 | 3.356 | 1.5 | 18 | 53 | 58.512 | 5.472 | Australia | 65.180 | invertebrates | 12 | Sedentary | 3.397 | 0.148 |
| *Anthochaera chrysoptera* | 14.413 | 7.368 | 4.6 | 35.4 | 6.100 | 3.492 | 0 | 13.8 | 39 | 57.245 | 8.313 | Australia | 66.938 | plant/seed/nectar | 10 | Sedentary | 4.500 | 0.117 |
| *Philemon corniculatus* | 20.325 | 8.329 | 5.4 | 40.8 | 9.587 | 5.478 | 0 | 24.6 | 61 | 57.138 | 10.738 | Australia | 102.043 | omnivore | 10 | Sedentary | 4.678 | 0.119 |
| *Sphecotheres viridis* | 13.800 | 3.999 | 4.6 | 20.4 | 6.000 | 3.347 | 0 | 12 | 11 | 55.782 | 7.800 | Australia | 122.276 | plant/seed/nectar | 18 | Sedentary | 2.844 | 0.000 |
| *Cacomantis flabelliformis* | 18.400 | 8.465 | 6.2 | 36.2 | 9.730 | 5.333 | 1.5 | 21.5 | 20 | 54.808 | 8.670 | Australia | 49.825 | invertebrates | 11 | Sedentary | 3.346 | 0.000 |
| *Dicrurus bracteatus* | 22.133 | 7.902 | 10.9 | 31 | 13.933 | 7.374 | 0 | 23 | 9 | 52.925 | 8.200 | Australia | 83.890 | invertebrates | 14 | Sedentary | 4.683 | 0.096 |
| *Troglodytes aedon* | 12.908 | 5.363 | 4.97 | 27.69 | 6.748 | 3.523 | 0 | 17.75 | 43 | 52.209 | 6.160 | USA | 10.925 | invertebrates | 9 | Sedentary | 4.712 | 0.000 |
| *Malurus cyaneus* | 12.770 | 5.994 | 1.8 | 31.3 | 6.151 | 3.126 | 1 | 15.4 | 89 | 50.831 | 6.619 | Australia | 10.415 | invertebrates | 10 | Sedentary | 3.382 | 0.073 |
| *Pardalotus punctatus* | 9.383 | 2.007 | 6.9 | 12.9 | 4.333 | 1.868 | 0.7 | 5.7 | 6 | 43.115 | 5.050 | Australia | 8.788 | invertebrates | 14 | Sedentary | 4.118 | 0.123 |
| *Larus dominicanus* | 67.869 | 34.514 | 27.7 | 146.2 | 27.123 | 11.022 | 12.6 | 44.8 | 13 | 40.637 | 40.746 | Australia | 970.715 | vertebrates | 15 | Sedentary | 3.297 | 0.019 |
| *Corvus corax* | 53.975 | 32.125 | 16.33 | 126.38 | 10.952 | 13.136 | 0.71 | 47.57 | 11 | 119.948 | 43.024 | USA | 1012.485 | vertebrates | 69 | PartiallyMigratory | 4.822 | 0.119 |
| *Falco cenchroides* | 64.343 | 49.145 | 7.7 | 165 | 41.714 | 45.368 | 0 | 143 | 14 | 108.758 | 22.629 | Australia | 171.485 | invertebrates | 18 | PartiallyMigratory | 5.644 | 0.008 |
| *Elanus axillaris* | 59.260 | 35.964 | 19 | 97.9 | 18.000 | 18.897 | 3.8 | 49 | 5 | 104.986 | 41.260 | Australia | 267.800 | vertebrates | 4 | PartiallyMigratory | 2.781 | 0.002 |
| *Molothrus ater* | 22.973 | 14.748 | 6.7 | 52.1 | 6.967 | 6.942 | 0 | 28.6 | 15 | 99.647 | 16.007 | USA | 41.405 | plant/seed/nectar | 17 | PartiallyMigratory | 3.871 | 0.175 |
| *Anthochaera carunculata* | 13.915 | 8.507 | 5.4 | 30.8 | 6.808 | 5.669 | 0.8 | 17.5 | 13 | 83.269 | 7.108 | Australia | 104.310 | plant/seed/nectar | 13 | PartiallyMigratory | 5.213 | 0.119 |
| *Acridotheres tristis* | 23.343 | 13.910 | 8.6 | 75.4 | 11.873 | 9.655 | 0.8 | 40 | 37 | 81.321 | 11.470 | Australia | 114.965 | omnivore | 6 | PartiallyMigratory | 4.120 | 0.084 |
| *Thryomanes bewickii* | 12.557 | 7.935 | 1.5 | 34.6 | 8.140 | 5.984 | 0 | 31.5 | 68 | 73.509 | 4.416 | USA | 9.900 | invertebrates | 8 | PartiallyMigratory | 3.903 | 0.012 |
| *Colaptes auratus* | 32.856 | 19.118 | 5.4 | 80.1 | 16.632 | 12.050 | 1.42 | 55.4 | 20 | 72.451 | 16.225 | USA | 131.066 | invertebrates | 13 | PartiallyMigratory | 4.697 | 0.155 |
| *Carpodacus mexicanus* | 17.508 | 9.399 | 3.8 | 50 | 9.746 | 6.773 | 0 | 36.1 | 85 | 69.495 | 7.762 | USA | 21.400 | plant/seed/nectar | 12 | PartiallyMigratory | 3.510 | 0.144 |
| *Corvus brachyrhynchos* | 36.401 | 15.468 | 14.3 | 91.59 | 21.684 | 14.476 | 0.71 | 85.2 | 43 | 66.758 | 14.717 | USA | 450.880 | omnivore | 41 | PartiallyMigratory | 5.035 | 0.132 |
| *Phalacrocorax carbo* | 56.277 | 26.435 | 15.4 | 115.4 | 33.229 | 21.705 | 6.2 | 84.6 | 31 | 65.320 | 23.048 | Australia | 2364.485 | vertebrates | 27 | PartiallyMigratory | 8.564 | 0.017 |
| *Oriolus sagittatus* | 16.080 | 8.319 | 3 | 38.5 | 9.523 | 5.946 | 0 | 20.8 | 30 | 62.436 | 6.557 | Australia | 95.900 | plant/seed/nectar | 12 | PartiallyMigratory | 3.507 | 0.177 |
| *Sturnus vulgaris* | 27.261 | 13.624 | 5.68 | 72.42 | 12.828 | 7.940 | 1 | 34.6 | 48 | 61.894 | 14.433 | Australia/USA | 77.408 | omnivore | 23 | PartiallyMigratory | 5.651 | 0.142 |
| *Cygnus atratus* | 103.900 | 45.705 | 15.4 | 181.3 | 57.626 | 32.927 | 2.8 | 133.1 | 19 | 57.139 | 46.274 | Australia | 5639.580 | plant/seed/nectar | 40 | PartiallyMigratory | 3.288 | 0.000 |
| *Euphagus cyanocephalus* | 22.329 | 5.125 | 16.8 | 32.8 | 15.386 | 8.692 | 0 | 26.9 | 7 | 56.494 | 6.943 | USA | 62.565 | invertebrates | 13 | PartiallyMigratory | 3.972 | 0.165 |
| *Larus delawarensis* | 54.843 | 20.904 | 20.6 | 95.6 | 22.457 | 12.353 | 0.7 | 46.3 | 21 | 55.005 | 32.386 | USA | 517.405 | vertebrates | 32 | PartiallyMigratory | 4.899 | 0.135 |
| *Pachycephala pectoralis* | 14.994 | 6.967 | 5.4 | 26.9 | 7.947 | 4.278 | 0 | 15.4 | 17 | 53.835 | 7.047 | Australia | 31.530 | invertebrates | 18 | PartiallyMigratory | 4.879 | 0.073 |
| *Elseyornis melanops* | 40.559 | 15.633 | 12.3 | 68.5 | 23.772 | 10.791 | 1 | 49.2 | 32 | 45.392 | 16.788 | Australia | 32.250 | invertebrates | 7 | PartiallyMigratory | 4.870 | NA |
| *Sturnella neglecta* | 57.214 | 21.363 | 26.98 | 104.37 | 35.086 | 13.835 | 14.2 | 51.83 | 12 | 39.433 | 22.128 | USA | 98.880 | omnivore | 10 | PartiallyMigratory | 3.000 | 0.184 |
| *Buteo lineatus* | 40.524 | 30.034 | 7.7 | 84.49 | 32.830 | 30.557 | 0 | 77.39 | 5 | 93.075 | 7.694 | USA | 609.860 | vertebrates | 26 | Migratory | 2.872 | 0.019 |
| *Lichenostomus chrysops* | 9.728 | 5.294 | 3.8 | 22.3 | 4.669 | 4.203 | 0 | 15.4 | 29 | 90.023 | 5.059 | Australia | 17.300 | plant/seed/nectar | 13 | Migratory | 4.266 | 0.215 |
| *Accipiter cooperii* | 26.989 | 9.642 | 16.9 | 41.18 | 10.009 | 8.385 | 1.42 | 21.5 | 7 | 83.777 | 16.980 | USA | 440.835 | vertebrates | 20 | Migratory | 3.828 | 0.002 |
| *Turdus migratorius* | 22.488 | 12.388 | 4.2 | 63 | 10.470 | 7.911 | 0 | 51.2 | 92 | 75.557 | 12.018 | USA | 78.642 | omnivore | 17 | Migratory | 4.538 | 0.105 |
| *Dendroica petechia* | 13.176 | 6.519 | 4.2 | 31.5 | 6.256 | 4.495 | 0 | 17.6 | 51 | 71.849 | 6.920 | USA | 10.220 | invertebrates | 11 | Migratory | 4.345 | 0.024 |
| *Mimus polyglottos* | 21.140 | 8.792 | 9.94 | 44.73 | 12.116 | 8.551 | 0 | 38.34 | 28 | 70.581 | 9.024 | USA | 48.500 | omnivore | 20 | Migratory | 4.165 | 0.071 |
| *Calidris minutilla* | 23.246 | 17.069 | 8.1 | 96.3 | 9.164 | 5.707 | 1.9 | 26.5 | 39 | 62.281 | 14.082 | USA | 23.165 | invertebrates | 16 | Migratory | 6.754 | 0.000 |
| *Polioptila caerulea* | 19.150 | 16.145 | 7.7 | 50.8 | 8.000 | 4.684 | 3.8 | 16.9 | 6 | 58.556 | 11.150 | USA | 5.963 | invertebrates | 4 | Migratory | 4.467 | 0.000 |
| *Regulus calendula* | 10.102 | 4.032 | 3.8 | 21.3 | 4.180 | 2.297 | 0.8 | 12.07 | 46 | 54.949 | 5.922 | USA | 6.383 | invertebrates | 6 | Migratory | 1.895 | 0.073 |
| *Falco sparverius* | 46.600 | 22.818 | 21.5 | 72.42 | 23.043 | 12.292 | 4.26 | 42.6 | 6 | 53.343 | 23.557 | USA | 115.055 | invertebrates | 17 | Migratory | 5.963 | 0.008 |
| *Pluvialis squatarola* | 63.454 | 29.837 | 15.4 | 159.6 | 35.818 | 18.586 | 8.8 | 107.4 | 67 | 51.891 | 27.636 | Australia/USA | 237.000 | invertebrates | 26 | Migratory | 3.506 | 0.069 |
| *Limosa fedoa* | 45.170 | 23.082 | 12.5 | 150 | 17.448 | 8.517 | 3.7 | 36.8 | 46 | 48.814 | 27.722 | USA | 363.635 | invertebrates | 14 | Migratory | 3.785 | 0.000 |
| *Junco hyemalis* | 15.008 | 6.537 | 3.55 | 32.66 | 8.494 | 3.941 | 0 | 18.5 | 44 | 46.395 | 6.513 | USA | 19.488 | plant/seed/nectar | 11 | Migratory | 2.989 | 0.175 |
| *Geothlypis trichas* | 12.388 | 4.636 | 4.26 | 23.8 | 8.644 | 3.923 | 1.5 | 16.9 | 38 | 45.385 | 3.744 | USA | 9.603 | invertebrates | 12 | Migratory | 3.448 | 0.000 |
| *Todiramphus sanctus* | 31.736 | 13.767 | 7.7 | 54.6 | 21.018 | 8.069 | 6.9 | 34.6 | 11 | 38.391 | 10.718 | Australia | 53.455 | invertebrates | 8 | Migratory | 7.577 | 0.008 |
| *Myiarchus cinerascens* | 27.375 | 10.748 | 13.1 | 49.2 | 16.150 | 5.177 | 10 | 24.6 | 12 | 32.059 | 11.225 | USA | 28.200 | invertebrates | 12 | Migratory | 4.062 | NA |
| *Agelaius phoeniceus* | 29.410 | 9.990 | 11.8 | 43.7 | 15.487 | 4.646 | 10.1 | 23.5 | 7 | 29.997 | 13.923 | USA | 52.390 | omnivore | 20 | Migratory | 2.206 | 0.175 |
| *Charadrius vociferus* | 37.820 | 13.440 | 19.9 | 57.4 | 29.100 | 6.852 | 17.6 | 35.3 | 5 | 23.545 | 8.720 | USA | 95.358 | invertebrates | 11 | Migratory | 4.864 | 0.058 |

Table S3. Results of an alternative model explaining plasticity in escape behaviour in birds as a function of starting distance, the number of observations (FID count), habitat and diet breadth, migratory behaviour (migratory, partially migratory and sedentary birds), body mass, life span and diet type of species, considering the country where data were collected. The model is a generalized linear model based on the dataset used in previous analyses, after filtering two species recorded simultaneously in both countries. Model is based on 3699 observations of FID collected in USA (1240 records) and Australia (2359 records) for 93 bird species. The table shows the values of estimates, the lower (2.5%) and upper (97.5%) limits of confidence intervals, standard error (SE), t and p values. Significant variables are highlighted in bold.

| **Variable/Model** | **Estimate** | **2.50%** | **97.50%** | **SE** | **t value** | **p** |
| --- | --- | --- | --- | --- | --- | --- |
| Intercept | 58.810 | 35.223 | 82.401 | 12.040 | 4.887 | < 0.001 |
| Starting distance (mean) | -0.223 | -0.574 | 0.128 | 0.179 | -1.245 | 0.217 |
| FID (count) | -0.033 | -0.163 | 0.097 | 0.067 | -0.496 | 0.621 |
| Habitat breadth | 0.130 | -3.118 | 3.378 | 1.657 | 0.078 | 0.938 |
| Diet breadth | -50.770 | -150.833 | 49.296 | 51.050 | -0.994 | 0.323 |
| **Migration: Partially migratory** | 18.130 | 0.444 | 35.806 | 9.021 | 2.009 | 0.048 |
| **Migration: Sedentary** | 21.670 | 5.007 | 38.341 | 8.504 | 2.549 | 0.013 |
| Body mass | -0.001 | -0.008 | 0.006 | 0.004 | -0.185 | 0.854 |
| Life span (max) | 0.226 | -0.114 | 0.565 | 0.173 | 1.302 | 0.197 |
| Diet: Omnivore | -0.705 | -15.606 | 14.196 | 7.603 | -0.093 | 0.926 |
| Diet: Plant/seed/nectar | 7.446 | -6.331 | 21.223 | 7.029 | 1.059 | 0.293 |
| Diet: Vertebrates | 14.000 | -6.852 | 34.859 | 10.640 | 1.316 | 0.192 |
| Country: USA | 4.217 | -9.001 | 17.434 | 6.744 | 0.625 | 0.534 |

Figure S1. Association between behavior plasticity (coefficient of variation of FID) and no. observations (FID count). The point size reflects the species body mass. Blue line is the linear regression while envelopes around line are 95% confidence interval.


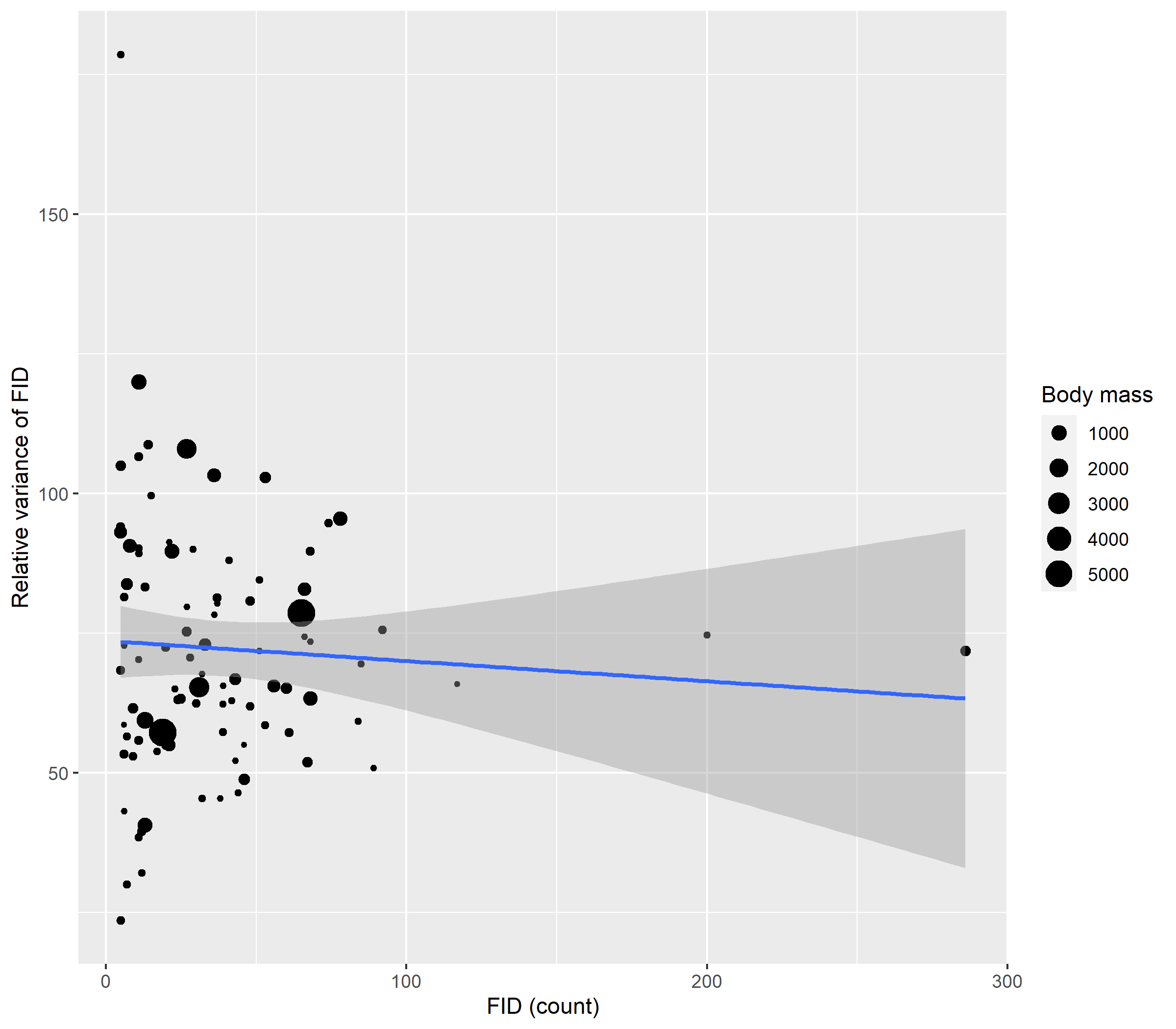


Figure S2. Avian phylogeny and species behavior plasticity (coefficient of variation of FID). The horizontal green bar indicates plasticity in escape behaviour.


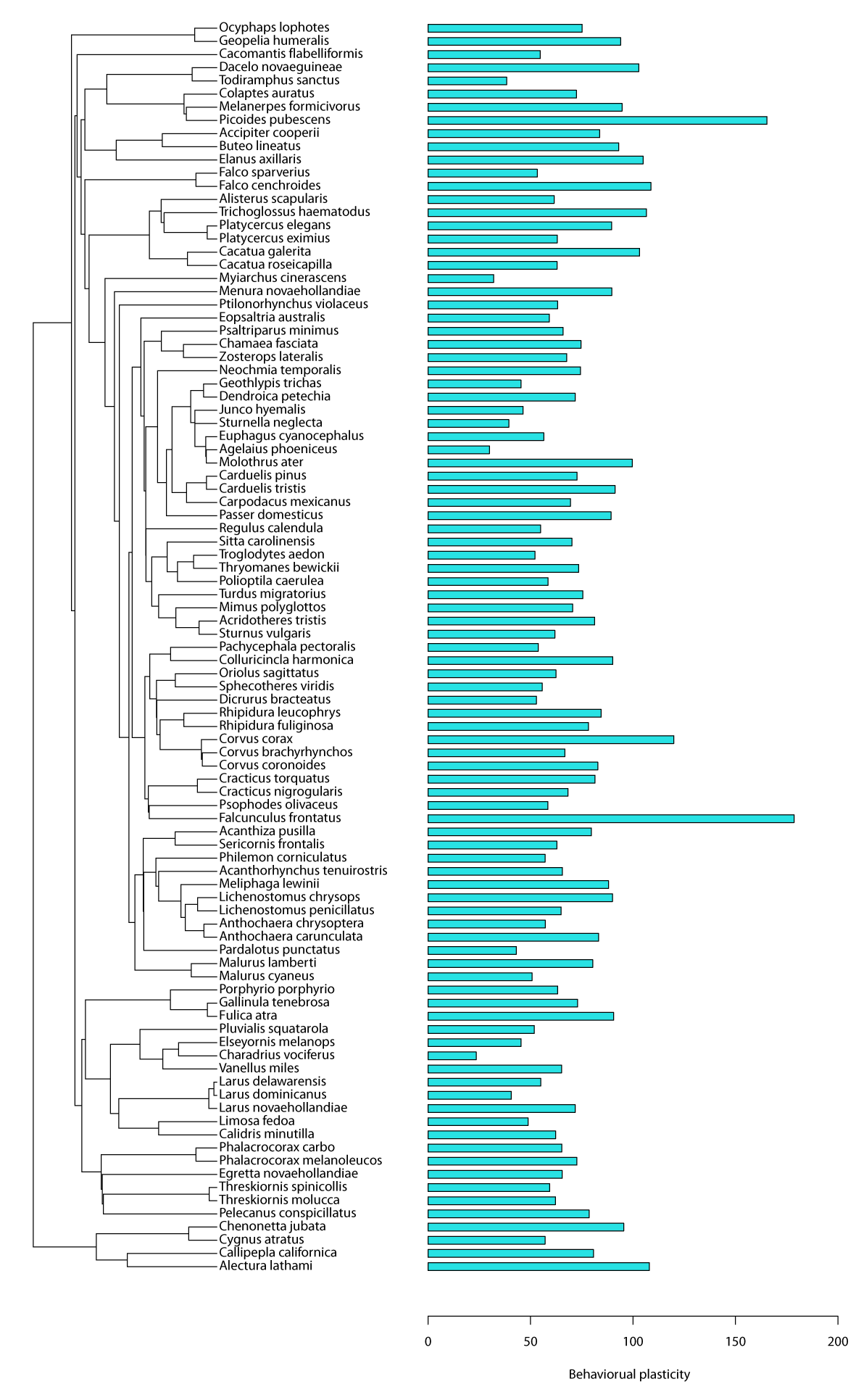


Figure S3. Association between behavior plasticity (coefficient of variation of FID) and diet breadth. The point size reflects the species body mass, while colours highlight the different type of diet of species. Blue lines are the linear regressions while envelopes around line are 95% confidence interval.


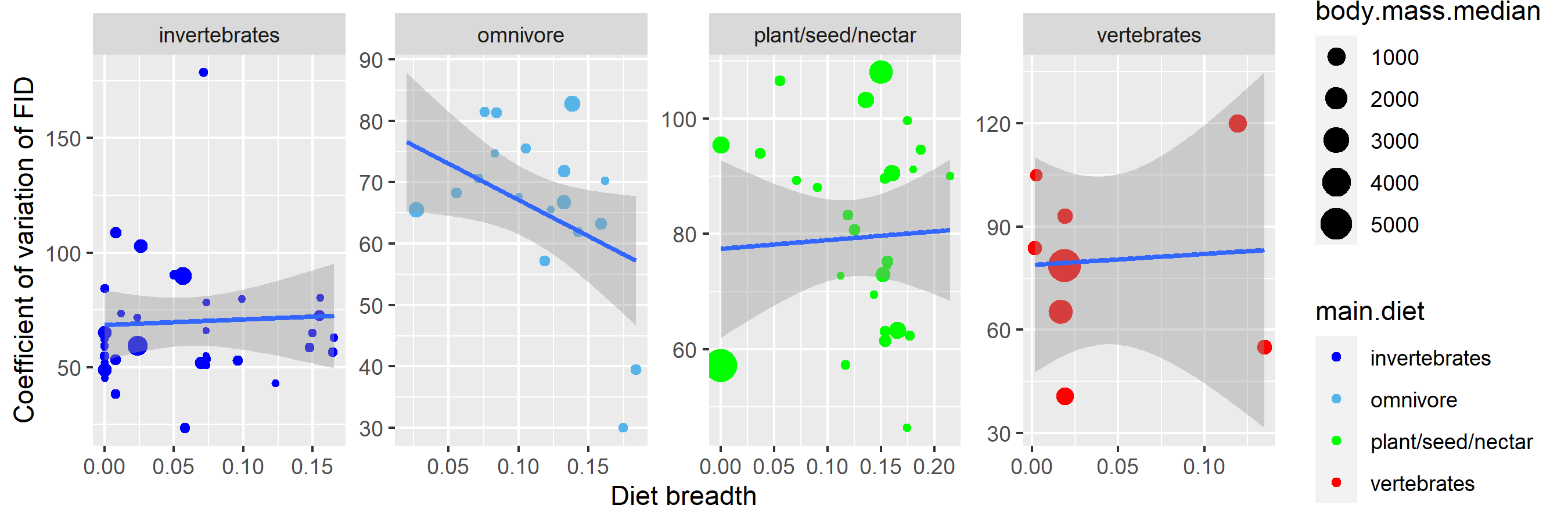


Figure S4. Association between behavior plasticity (coefficient of variation of FID) and habitat breadth. The point size reflects the species body mass, while colours highlight the different type of diet of species. Blue lines are the linear regressions while envelopes around line are 95% confidence interval.


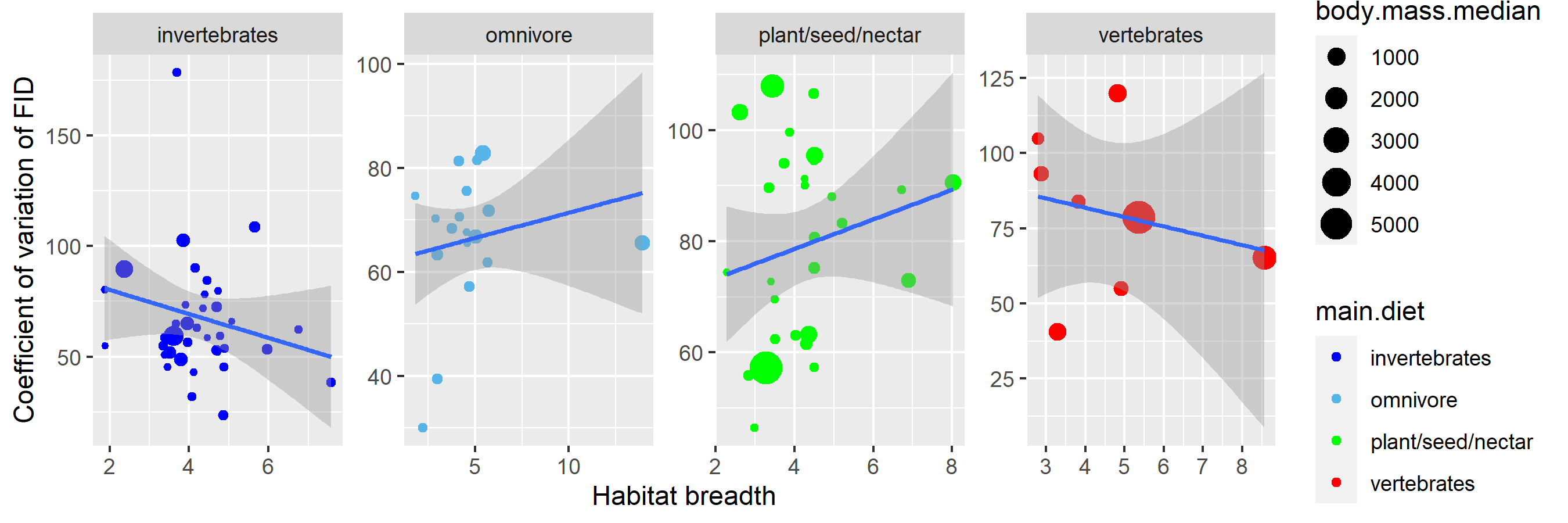


Figure S5. Association between behavior plasticity (coefficient of variation of FID) and life span (max). The point size reflects the species body mass, while colours highlight the different type of diet of species. Blue lines are the linear regressions while envelopes around line are 95% confidence interval.


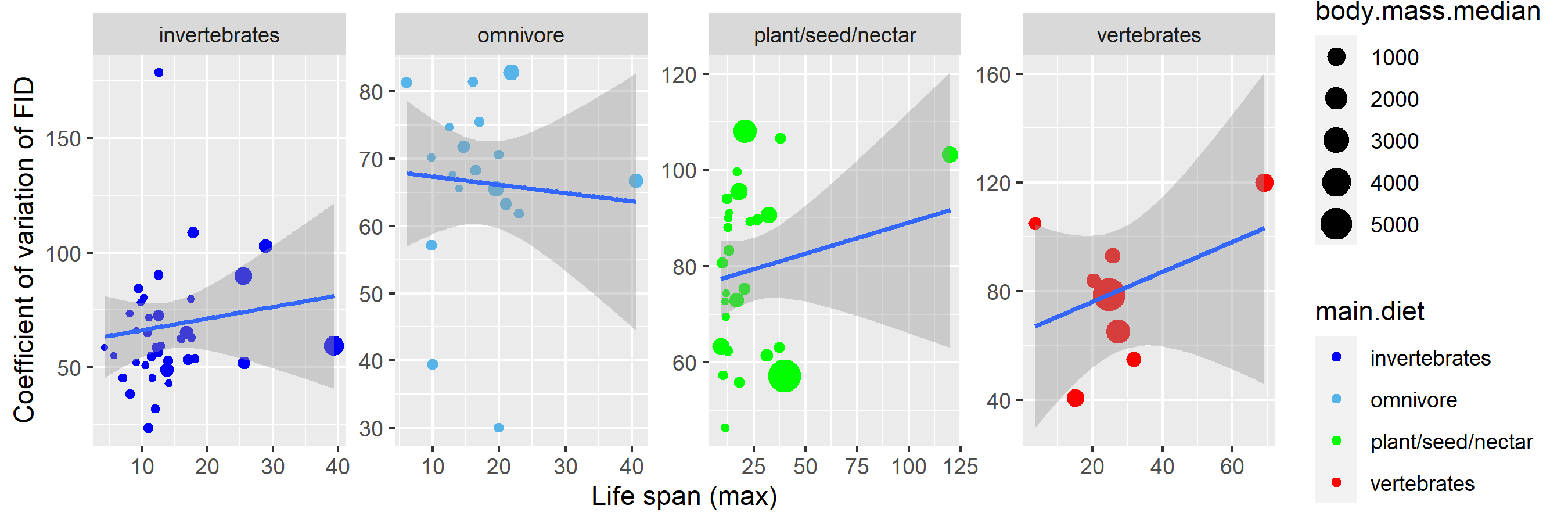

Supplement: Supplementary file 1 — Supplementary Information. [file 41598_2022_9834_MOESM1_ESM.docx]
